# The VEGF/VEGFR Axis Revisited: Implications for Cancer Therapy

**DOI:** 10.3390/ijms232415585

**Published:** 2022-12-09

**Authors:** Peace Mabeta, Vanessa Steenkamp

**Affiliations:** 1 Department of Physiology, Faculty of Health Sciences, University of Pretoria, Gezina 0031, South Africa; 2 Department of Pharmacology, Faculty of Health Sciences, University of Pretoria, Gezina 0031, South Africa

**Keywords:** full-length VEGF, VEGF_xxxa_, VEGF_xxxb_, VEGFR, cancer, angiogenesis, alternative splicing

## Abstract

The vascular endothelial growth factor (VEGF)/vascular endothelial growth factor receptor (VEGFR) axis is indispensable in the process of angiogenesis and has been implicated as a key driver of tumor vascularization. Consequently, several strategies that target VEGF and its cognate receptors, VEGFR-1 and VEGFR-2, have been designed to treat cancer. While therapies targeting full-length VEGF have resulted in an improvement in both overall survival and progression-free survival in various cancers, these benefits have been modest. In addition, the inhibition of VEGFRs is associated with undesirable off-target effects. Moreover, VEGF splice variants that modulate sprouting and non-sprouting angiogenesis have been identified in recent years. Cues within the tumor microenvironment determine the expression patterns of these variants. Noteworthy is that the mechanisms of action of these variants challenge the established norm of VEGF signaling. Furthermore, the aberrant expression of some of these variants has been observed in several cancers. Herein, developments in the understanding of the VEGF/VEGFR axis and the splice products of these molecules, as well as the environmental cues that regulate these variants are reviewed. Furthermore, strategies that incorporate the targeting of VEGF variants to enhance the effectiveness of antiangiogenic therapies in the clinical setting are discussed.

## 1. Introduction

In the adult, the vascular endothelium is relatively quiescent and vessel formation is restricted to processes such as wound healing. However, aberrant activation of the vasculature occurs in various pathological conditions. Almost a century ago, Ide et al. (1939) observed that the aggressive growth of transplanted tumors was characterized by increased vascularization [1]. In 1975, Folkman postulated that for a tumor to grow beyond a critical size of 1–2 mm it needs to form new vessels mainly through the process of angiogenesis [2]. This process, also known as sprouting angiogenesis, entails the formation of new vessels from an already existing microvasculature and occurs under various physiological conditions to support growth and tissue repair. The remodeling of the newly formed vessels is mainly accomplished through non-sprouting angiogenesis [3,4,5]. Interestingly, tumors use the same process to support their own survival and progression. In the normal physiological setting, the balance between angiogenesis inhibitors and stimulators is intricately controlled [3,4]. On the flip side, the loss of balance between pro- and anti-angiogenic molecules promotes the transition from dormancy to malignancy [3,5,6]. As a result, research has focused on identifying factors that regulate angiogenesis and on understanding their behavior in the tumor setting [7].

Over the last four decades, significant advances were made in delineating the interplay between pro- and anti-angiogenic factors that foster an environment that favors the angiogenic phenotype. In 1979, a molecule that promotes angiogenesis and vessel permeability, vascular permeability factor (VPF), was identified [8]. Ferrara and colleagues later isolated a potent endothelial cell (EC) mitogen, vascular endothelial growth factor (VEGF), from the conditioned medium of cultured bovine pituitary follicular cells [9]. It subsequently became apparent from sequencing studies that VPF and VEGF were the same protein [10]. Since the sequencing of VEGF, other structurally related dimeric proteins have been identified, and these constitute the vascular endothelial growth factor family. Of the identified angiogenic molecules, VEGF has been the most studied due to its critical role in the regulation of physiological and pathological angiogenesis [11,12].

## 2. Vascular Endothelial Growth Factor in Physiology and Disease

In mammals, the VEGF family of proteins is composed of VEGF-A, VEGF-B, VEGF-C, VEGF-D, and placental growth factor (PlGF) [13]. The VEGF-A protein (referred to as VEGF in this review) is a 45-kDa homodimeric glycoprotein and is the best characterized of the VEGF family members [14]. The main stimulus of VEGF expression is hypoxia, which promotes the binding of hypoxia-inducible factor-1α (HIF-1α) to the hypoxia response element (HRE) in the VEGF promoter, resulting in the increased expression of the growth factor [14,15].

### 2.1. The VEGF in Physiological Angiogenesis

VEGF plays an important role in vasculogenesis, the de novo formation of a primordial vascular structure from endothelial precursor cells that occurs mainly during embryonic development [16,17]. The ligand is further required for angiogenesis during embryonic and postnatal development, although in the adult it is restricted to instances such as wound repair and the female reproductive cycles [14,18]. The main receptors for VEGF are vascular endothelial growth factor receptor-1 (VEGR-1) or fms-like tyrosine kinase-1 (Flt-1) and VEGF receptor-2 (VEGFR-2), also known as kinase insert domain-containing receptor (KDR) [13,14]. The exact mechanism of VEGFR-1 signaling is not entirely understood, however, it seems to function as a decoy receptor for VEGF [14,19]. Additionally, VEGFR-1 is the exclusive receptor for other VEGF family members, namely, VEGF-B and PlGF [18,20]. The receptor is essential in hematopoiesis, in the activation of matrix metalloproteinases (MMPs), as well as in the migration of monocytes and other immune cells into the tumor microenvironment (TME) [13,14,20]. On the other hand, VEGFR-2 is important for vasculogenesis and angiogenesis as it promotes both processes through several mechanisms [14,18]. The binding of VEGF to VEGFR-2 results in the activation of endothelial nitric oxide synthase (eNOS) as well as inducible nitric oxide synthase (iNOS) through the nitric oxide synthase (NOS) pathway [21]. This signaling pathway leads to the downstream release of vasodilators such as nitric oxide (NO) and a subsequent increase in vessel permeability [22]. The binding of VEGF to VEGFR-2 can also activate phoshotidylinositol-3 kinase (PI3k), resulting in the downstream activation of protein kinase B (PKB), which in turn promotes EC survival, proliferation, and tube formation [23,24]. In addition to the above-mentioned effects, VEGF activates focal adhesion kinase (FAK), which induces cell migration via paxillin, and thus also promotes angiogenesis [25]. Of note is that VEGFR-2 binds to VEGF with an affinity that is approximately 10-fold lower than that for VEGFR-1. Nonetheless, VEGFR-2 presents a greater signaling activity and as a result, the mitogenic effects of VEGF are mainly mediated via VEGFR-2 [12,14]. In addition, VEGFR-2 plays a key role in mediating VEGF-induced EC migration and vessel permeability, whereas VEGFR-1 exhibits a weak or undetectable response in this regard [14,18].

### 2.2. VEGF/VEGFR-2 Signaling in Tumor Angiogenesis

The upregulation of VEGF has been demonstrated in various tumors, both benign and malignant, including juvenile hemangioma, glioblastoma multiforme, melanoma, breast, lung, head and neck, ovarian, gastrointestinal tract, and renal carcinomas [2,15,26,27,28]. In a subset of melanoma patients, increased VEGF levels were found to correlate with tumor thickness, while in another study it was shown that VEGF confers increased mitogenic potential in stromal cells, including immune cells, isolated from human tumor biopsies [28,29,30]. In addition, there is a positive relationship between mutations in VEGFR-2 and tumor progression [30]. In juvenile hemangioma, a C482R mutation in VEGFR-2 results in the amplification of VEGF/VEGFR-2 signaling and an increase in angiogenesis, while in neoplasms such as melanoma, VEGF upregulation is associated with disease progression [30]. Thus, in the tumor setting, the end result of the activation of the VEGF/VEGFR signaling axis is an increase in vessel density, invasiveness, immune escape, and in some instances, an enhanced metastatic capacity [31]. Not surprisingly, drugs have been developed to target the VEGF/VEGFR axis, however, these treatments have had limited success. Recent studies have revealed the presence of variants derived from VEGF splicing that may influence signaling through this axis.

## 3. Alternative Splicing of VEGF and Angiogenesis

Alternative splicing involves the removal of introns from pre-messenger ribonucleic acid (pre-mRNA), with the remaining exons being connected to each other in different combinations to form mRNAs [32,33]. When dysregulated, the generation of variants that promote tumorigenesis may occur [34,35,36,37,38,39,40,41,42]. Moreover, some of the variants formed from the alternative splicing of full-length or total VEGF are linked to impaired angiogenesis and tumor progression [35,36]. The discovery of several VEGF isoforms with distinct functions has revealed that the physiology of VEGF is more complex than previously thought [37]. Approximately sixteen isoforms have been identified in humans thus far, and six (VEGF-A_111_, VEGF_121_, VEGF_145_, VEGF_165_, VEGF_181_, VEGF_206_) have been studied extensively in terms of structure and biochemical properties [37]. In mice, VEGF_120_, VEGF_164_, and VEGF_188_ are abundant [37,38]. The transcripts are assigned numerical values based on the number of amino acids present [38]. The alternative splicing of VEGF is stimulated by several factors including pH, hypoxia, and nutrient levels [39]. Acidic environments (~pH 5.5) have been shown to induce alternative splicing of VEGF, resulting in the formation of mainly VEGF_121a_, followed by VEGF_165a_. The increase in VEGF_121a_ is often associated with p38 activation [39]. Other variants, namely, VEGF_145a_ and VEGF_189_ have merely shown slight increases in an acidic pH. In hypoxic conditions, there is a tilt toward the formation of VEGF_165a_ and VEGF121a [39]. This shift has been observed in both cancer cell lines and human tumors, although the pattern of expression of these variants depends on the cancer type [39].

The bioavailability of VEGF variants is dependent on their chemical structure and properties. For instance, VEGF_111a_ and VEGF_121a_ do not bind to matrix glycoproteins and therefore can diffuse easily and are thus readily available. On the other hand, VEGF_145a_, VEGF_189a_, and VEGF_206a_ bind to heparin and heparin sulfate proteoglycans on the cell surface and in the extracellular matrix (ECM) with the strongest affinity compared to the other isoforms, and as a result, they have the lowest bioavailability [40]. It is important to note that VEGF_145a_ and VEGF_206_ appear to be rare compared to other variants [41]. VEGF_165a_ binds to heparin proteoglycans with intermediate affinity, exhibits moderate to high bioavailability, and is a more potent inducer of angiogenesis than the other isoforms [41].

### 3.1. VEGF_111a_ and VEGF_121a_

**Figure 1:**
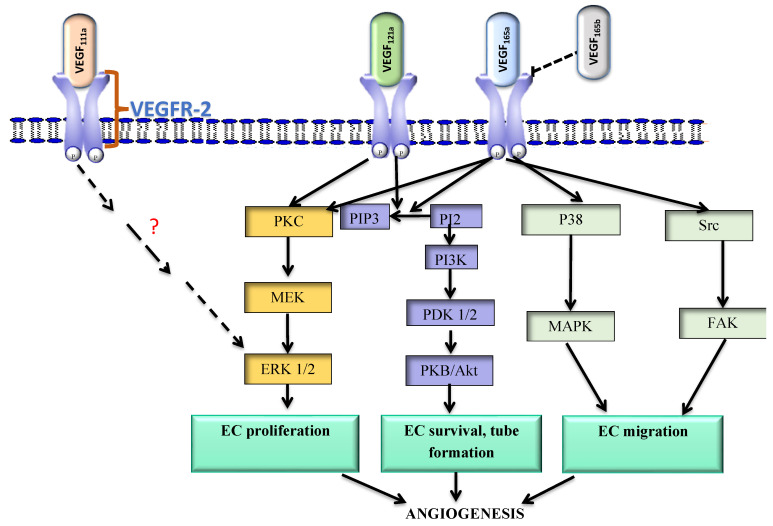
Signaling pathways activated by VEGF isoforms that promote angiogenesis. VEGF_xxxb_ has an inhibitory effect on the VEGF_xxxa_-VEGFR-2 complex.

VEGF_111a_ and VEGF_121a_ exist as highly soluble molecules and are the most bioavailable VEGF isoforms identified to date. VEGF_111a_ was identified in 2007 and has since been shown to be a potent EC mitogen and an inducer of angiogenesis in vivo [42]. It can bind to VEGFR-1 and VEGFR-2, although it is the mechanism involving the latter receptor that has received attention. VEGF_111_’s mechanism of action through VEGFR-2 appears to be exerted via the protein kinase C (PKC)-extracellular signal-regulated kinase (ERK) 1/2 pathway (Figure 1). In aortic ECs and human vein umbilical endothelial cells (HUVECs) the isoform has been shown to induce the phosphorylation of VEGFR-2, leading to the downstream activation of ERK 1/2 [43]. Although VEGF_111_ binds to VEGFR-2, it does not bind sufficiently to the neuropilin-1 (NRP-1) co-receptor to form the NRP-1/VEGFR-2 complex, thus its angiogenic effects might not be as strong [43]. VEGF_111a_ is abundant in the lungs and kidneys [43].

VEGF_121a_ can also bind to both VEGFR-1 and VEGFR-2, although there is a paucity of data on its binding to the former receptor. The binding of the ligand to VEGFR-2 activates the PI3k signaling pathway (Figure 1), resulting in endothelial cell survival [44]. It also promotes lymphatic vessel formation, although there is limited data on its mechanism [44,45]. As well, VEGF_121a_ activates mitogen-activated protein kinase kinase (MEK) and ERK 1/2, leading to the formation of EC tubes and their maturation (Figure 1). It is a potent inducer of tumorigenesis in experimental models. In mouse xenografts of renal cell carcinoma (RCC) and non-small cell lung carcinoma (NSCLC), VEGF_121a_ together with another variant, VEGF_165a,_ were found to promote angiogenesis [46]. However, an investigation of the effects of this isoform on vascular physiology is necessary to better understand its contribution to tumor angiogenesis and its possible interaction with other isoforms such as VEGF_165a_, which is regarded as the prototype of VEGF.

### 3.2. VEGF_165a_

VEGF_165a_ is a moderately diffusible isoform and approximately 60% of the protein is associated with both the cell surface and the ECM [41]. It can bind to VEGFR-1 and VEGFR-2, as well as to the co-receptor NRP-1 [41,42]. It induces VEGFR-2 phosphorylation leading to signal transduction mainly via protein kinase B (PKB) and ERK 1/2 (Figure 1). The activation of PKB leads to EC survival, while the activation of the ERK 1/2 pathway promotes EC proliferation and regulates vessel diameter [40,41,42]. In vitro studies conducted using Chinese hamster ovary (CHO) cells have shown that VEGF_165a_ induces the activation of p38 and mitogen-activated protein kinase (MAPK), resulting in the reorganization of the actin cytoskeleton and ultimately promoting cell migration (Figure 1) [40,41,42]. As well, the downstream activation of focal adhesion kinase stimulates the migration of ECs [41,42]. In addition, when the VEGFR-2 co-receptor, NRP-1 is overexpressed, it potentiates the effects of VEGF_165a_, leading to an increase in the proliferative ability of ECs as well as their invasion [40,41,42,47]. In vivo, VEGF_165a_ is overexpressed in several cancers and similar to VEGF_111a_ and VEGF_121a_ promotes disease progression [47,48,49,50].

### 3.3. VEGF_165b_

The detection of VEGF_165b_ was initially described by Bates and colleagues following the observation of a reduced expression of the protein in renal cancer tissue when compared to non-cancerous tissue [51]. The observations were followed by several reports citing the identification of VEGF_165b_ in various tissues, including the skin [52,53,54]. Diverse findings have been reported on the functions of the VEGF_xxxb_ variants, with some reports indicating that VEGF-A_165b_ results in a far more reduced angiogenic effect when compared to VEGF_xxxa_, while other studies have reported that VEGF_165b_ inhibits angiogenesis [52,54,55]. Woolard and colleagues observed that VEGF_165b_ failed to induce the activation of VEGFR-2 in human microvascular endothelial cells [56]. The anti-angiogenic effects of VEGF_xxxb_ seem to emanate from its inhibition of VEGF_xxxa_’s interaction with VEGFR-2 [57]. The observations from the different studies on the effects of VEGF_xxxb_ may not necessarily be contradictory but might be due to the influence of the different tissue environments. With respect to the mechanism of the isoform, researchers have found that the binding of VEGF_165b_ to VEGFR-2 stimulates ERK 1/2 and PKB phosphorylation in ECs, although the induction of these pathways was considerably weak [58]. Interestingly, the prototype isoform, VEGF_165a_, was shown to stimulate mitogen-activated protein kinase (MAPK), while in the same cell line, VEGF_165b_ did not activate MAPK [59]. In addition, there was no hydrolysis of phosphoinositol 4,5-biphosphate (PIP2) observed downstream of VEGF_165b_-VEGFR-2 [59]. Moreover, Kawamura et al. noticed that VEGF_165b_ did not induce tube formation in embryonic stem cells or matrigel plugs and poorly induced VEGFR-2 phosphorylation at the Y1052 site [60]. Taken together, these observations indicate that the ligand has a markedly low ability to induce angiogenesis. Furthermore, a correlation was found between the binding affinity of VEGF_xxxb_ for NRP-1 and the inability of the ligand to induce angiogenesis [60]. Of note is that the VEGF isoforms, including VEGF_165b_, are expressed differentially in various cancers (Table 1), and in some instances, their expression appears to correlate with clinical outcomes [61,62,63]. In addition, the receptors through which these isoforms communicate can also undergo alternative splicing.

### 3.4. Alternative Splicing of VEGF Receptors

The VEGF receptor-1 exists in two isoforms that are derived from the alternative splicing of an mRNA sequence transcribed from a single gene [72]. The two isoforms are the transmembrane-bound protein, VEGFR-1, and a soluble polypeptide, sVEGFR-1 [72]. VEGF binding to the membrane-bound VEGFR-1 induces monocyte migration and is linked to the activation of MMPs [72,73]. An interesting observation is that the promoter region of the membrane-spanning VEGFR-1 has a HIF-1 consensus, and the receptor is thus responsive to hypoxic conditions [41,74]. Membrane VEGFR-1 appears to be an important link between tumor angiogenesis and immunity, considering that monocytes are not just involved in mediating immunity, but also secrete factors that promote angiogenesis. Then again, sVEGFR-1 can trap VEGF and lower the levels of the free form of this ligand, thus diminishing its ability to induce angiogenesis [75]. Furthermore, it is worth noting that the angiogenic effects of VEGFR-1 are weak compared to those induced via VEGFR-2.

Alternative splicing of VEGFR-2 yields a full-length receptor and a soluble form that contains only the extracellular domain, sVEGFR-2 [76,77]. However, the latter variant appears to play a more important role in the regulation of lymphangiogenesis rather than angiogenesis, although it has been detected in human umbilical vein endothelial cells (HUVECs) [76]. VEGFR-2 variants result from partial intron 13 retention. The translation product of the sVEGFR-2 mRNA is a protein with six (instead of seven) Ig-like domains which differ from the full-length VEGFR-2 in that it has a C-terminal sequence that is not found in the latter [77]. VEGF binding to the membrane-tethered VEGFR-2 isoform results in the phosphorylation of the receptor, leading to the activation of several signaling molecules, including phosphoinositide phospholipase C (PLCγ), phosphatidylinositol (3,4,5)-triphosphate (PIP3) and Ras [77,78]. PIP3 activates PKB, resulting in the promotion of cell survival and proliferation. Signaling through PLCγ and NO leads to vaso-permeability. In ECs, VEGFR-2 phosphorylation at Y801 activates the PI3k/PKB and eNOS pathways, while the phosphorylation of Y1059 (pY1059) leads to the flux of calcium which activates the MAPK pathway [21]. The phosphorylation of Y951 (pY951) is associated with cell motility, whereas pY1175 enables a binding site for PLCγ-l [21,77,78]. VEGF signaling through this VEGFR-2 isoform also induces iNOS, increasing the levels of this enzyme, and ultimately leading to increased vessel permeability [21,78]. sVEGFR-1 and sVEGFR-2 have been measured in blood samples of breast cancer patients receiving bevacizumab in combination with chemotherapy and both increased significantly following treatment [78]. However, the significance of this increase is not yet clear, and studies are needed to unravel the clinical implications of the levels of these soluble proteins. On the contrary, the chemistry and regulation of VEGF isoforms have been studied extensively.

### 3.5. Regulation of VEGF Splicing

Various factors regulate the generation of VEGF variants. In addition to environmental cues such as hypoxia and low pH, several kinases are involved in regulating VEGF splicing. The splicing of VEGF at the proximal splice site is regulated by serine/arginine-protein kinase 1(SRPK1) through the modulation of serine and arginine-rich splicing factor 1 (SRSF1) [79,80,81]. SRPK1 activation leads to the nuclear translocation of SRSF1 in a heat shock protein (HSp)90-dependent process [79]. SRSF1 in turn regulates the alternative splicing of various angiogenesis-promoting genes, namely, RON, TREAD1, and VEGF [79]. Several splice products formed from the proximal splicing such as VEGF_xxxa_, are stimulators of angiogenesis. Moreover, TREAD1 activates total VEGF and thus further contributes to the angiogenic process [79]. The distal splice site is modulated by the splice kinase CDC-like kinase 1 (Clk1) which regulates the splice factor SRSF6 [79]. The product of distal splicing, VEGF_xxxb_, appears to reduce angiogenesis. The SRFs that modulate VEGF splicing could potentially serve as targets for altering the splicing switch and restoring the VEGF_xxxa/xxxb_ ratio. The restoration of the ratio between VEGF_xxxa_ and VEGF_xxxb_ is of importance given the roles of these variants in the clinical outcomes of cancer patients.

## 4. Clinical Implications of VEGF Splice Products

**Figure 2:**
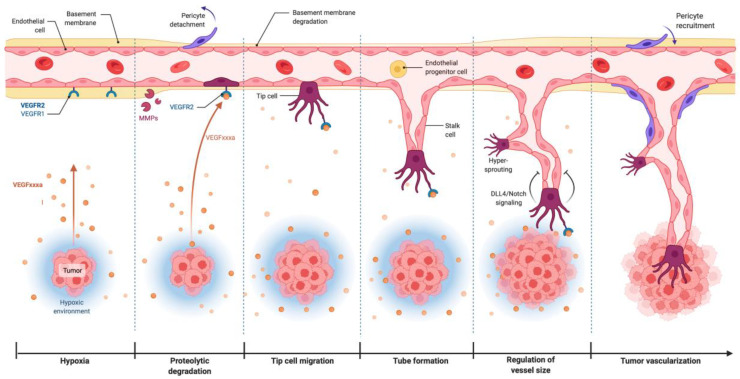
Tumor cells secrete VEGF_xxxa_, which initiates a series of steps that culminate in increased angiogenesis. Image was created using BioRender (2022).

VEGF_xxxa_ stimulates tumor angiogenesis, while VEGF_xxxb_ seems to suppress the process by limiting the binding of VEGF_xxxa_ to VEGFR-2 (Figure 2) [56,60]. As a nascent tumor grows, its nutrient and oxygen demand rise, leading to the increased secretion of total VEGF, which in turn is spliced to various isoforms depending on the pH in the TME and the degree of hypoxia [38,39]. VEGF_165a_ represents the predominant form in hypoxic conditions, and after binding to the VEGF receptor-2 on the surface of ECs, results in the activation of these cells and their secretion of various molecules, including proteolytic proteins [38,39,41]. Proteolysis of the basement membrane and ECM components by MMPs and the plasminogen activator (PA) system promotes the incursion of ECs into the tumor stroma (Figure 2) [12,18]. Tip cells lead to new sprouts and prepare the surrounding area for guidance cues. Stalk cells follow and support tip cells. The adhesion of the tip and stalk cells to the extracellular matrix is facilitated by integrins that are expressed by migrating ECs (Figure 2) [12,38,39]. Several signaling pathways including Delta-like ligand 4 (DLL4)-Notch signaling interact to regulate sprout formation. The tip cells anastomose with cells from adjacent sprouts to form vessel loops. The final and stabilizing step consists of the construction of adherent junctions and the basement membrane as well as the recruitment of pericytes [18]. These steps, which constitute the process of sprouting angiogenesis, lead to an increase in tumor vascularization.

The cleavage of full-length VEGF generates isoforms that are expressed differentially in various tissues and cancers [82,83]. In non-small cell lung cancer (NSCLC) VEGF_111a_ is overexpressed and is associated with an increase in the occurrence of metastasis [45]. The isoform is also highly expressed in breast and ovarian carcinomas, although no correlation has been found between its levels and patient outcome [84]. Another isoform, VEGF_121a_, has been shown to be elevated in prostate cancer when compared to normal prostate tissue [50]. Important to note is that in cancerous prostate tissue, elevated VEGF_121a_ levels are associated with cancer cell invasion and metastatic dissemination. The increased expression of the ligand in prostate cancer also correlates with hypoxia, which means oxygen deprivation may be an important driver of VEGF_121a_ overexpression and possibly angiogenesis in this neoplasm.

In human colorectal cancer, VEGF_121_ is highly expressed, and its expression is greater in patients exhibiting extensive infiltration of the lymph nodes by cancer cells [46]. VEGF_121_ is also highly expressed in breast cancer and is associated with increased angiogenesis in this neoplasm [39,84]. Although it is the predominant isoform in human breast cancer tissue, no association has been found between its expression levels and clinical outcome in breast cancer [84].

VEGF_165a_ is expressed in most cancers and is the predominant isoform [39]. In a previous study, it was detected in 70% of renal cell carcinoma (RCC) patients [46]. However, since the number of patients was not stipulated, the frequency of expression in RCC cannot be deduced from that study. In colorectal cancer, VEGF_165a_ is the predominantly expressed variant, and it correlates with lymph node infiltration [46]. Studies on cervical tissue specimens have revealed that VEGF_165a_ is overexpressed when compared to non-cancerous tissue isolated from the cervix [48]. Furthermore, the expression of the ligand correlates with lymph node metastasis. Similarly, in patients with renal squamous cell carcinoma (SCC) the overexpression of VEGF_165a_ is linked to disease recurrence and low disease-free survival while in esophageal cancer VEGF_165a_ expression correlates with microvessel density (MVD) [48,49]. Additionally, patients with various cancer overexpressing VEGF_165a_ have been reported to exhibit increased MVD and poor overall survival [49]. Interestingly, no such association has been observed between MVD and total VEGF in cancers such as melanoma and esophageal cancer [49]. This lack of correlation between total VEGF and MVD in some cancers which is observed with VEGF_xxxa_ may be due to the distinct and differing effects of VEGF_xxxb_ variants. It is also worth noting that it is not just the levels of total VEGF that plays a key role in regulating the angiogenic process, but also the balance in the levels of the variants. For instance, an increase in the ratio of VEGF_121a_ to VEGF_165-189a_ promotes angiogenesis in prostate cancer [50]. Studies have further shown that changes in the ratio of VEGF_165a_ to VEGF_165b_ contribute to disease progression in some cancers [52,83].

VEGF_xxxb_ has been detected in several tumors including melanomas that were growing in both the horizontal and vertical phases [61]. Of note is that the reduced expression of VEGF_xxxb_ correlated with the development of metastasis in melanoma patients [61]. The levels of VEGF_165b_ expression have been found to be lower in cancer tissue when compared to adjacent non-cancerous tissue [59,62]. Investigations of circulating levels of VEGF_165b_ in breast cancer patients revealed that the plasma levels of the ligand were significantly lower in cancer patients compared to healthy individuals [62]. Additionally, VEGF_165b_ levels increased following treatment and remained high even two years after chemotherapy [62]. However, no relationship was found between levels of this ligand and the tendency to relapse. VGEGF_165b_ levels were found to be elevated in 36% of patients with NSCLC and in 46% of lung adenocarcinoma patients [63]. Although no clinical significance has been attributed to the levels of the ligand either in lung adenocarcinomas or NSCLC, a shift in the ratio of VEGF_165a_ to VEGF_165b_ consistently correlated with lymph node metastasis in these patients [63]. VEGF_165a_ is not only the prototype for sprouting angiogenesis but has been shown to play a critical role in non-sprouting angiogenesis. The latter, also known as intussusceptive angiogenesis, involves the creation of vessels through the splitting of existing ones and is an alternative mechanism of vascularization that is used by various tumors [85]. Intussusception is also a mechanism used by tumors to escape anti-angiogenic therapy [85]. There is a correlation between intussusceptive angiogenesis and the development of resistance to angiogenesis inhibitors. This form of angiogenesis allows tumors to respond to their metabolic needs and to grow. Vascular bifurcation density analysis revealed that another isoform, VEGF_121_, is a potent inducer of intussusceptive angiogenesis [86]. Interestingly, while the administration of either of these isoforms leads to an increase in non-sprouting angiogenesis, their withdrawal results in a reduction in vessel branches and intussusceptive pruning [86]. The observations from the various studies highlight the importance of the different variants in tumor angiogenesis and the balance in VEGF_xxxa_ and VEGF_xxxb_, however, more work remains to be undertaken in order to determine the roles of the different variants in the prognosis of various cancers.

### Improving VEGF-Targeting Approaches

The observation that VEGF isoforms are associated with different neoplasms underscores a need to actively investigate therapeutic molecules that modulate them. The first anti-VEGF drug to be approved by the Food and Drug Association (FDA), bevacizumab, neutralizes total VEGF including the VEGF_xxxa_ and VEGF_xxxb_ isoforms by blocking their kinase domain binding sites [87]. Other anti-angiogenic drugs, cediranib, vandetanib, pazopanib, and sorafenib were also reported to inhibit the angiogenic effects of VEGF_165a_ [88]. However, given that these drugs are tyrosine kinase inhibitors (TKI’s), their effects could be due to their actions on VEGF receptors and not necessarily on the ligand. As a result, isoforms that signal through kinases inhibited by these drugs will have a diminished effect. Additionally, VEGF and its variants regulate VEGF receptor function. Full-length VEGF can upregulate its canonical receptors, VEGFR-1 and VEGFR-2 [89]. As well, the administration of VEGF_121a_ leads to an increase in the expression of VEGFR-1 in capillaries, while the presence of VEGF_165a_ promotes the co-expression of NRP1 and VEGFR-2 [90,91]. Findings from these studies show that the relationship between VEGF and its receptors is not simple, but rather intricate. The complex interaction between VEGF or its variants with VEGF receptors can potentiate angiogenesis, and as a result, more precise approaches are needed to subvert these interactions in a manner that is tumor specific. A plausible approach is the targeting of regulators associated with VEGF splicing, as well as the VEGF splice products which increase receptor activation and promote tumor vascularization. In experimental models, SRPKI was shown to be involved in the promotion of VEGF splicing, resulting in alterations in the VEGF_165a_/VEGF_165b_ ratio that favored increased VEGF_165a_ formation. Of interest is that Hulse and colleagues reported that the inhibition of SRPKI leads to a decrease in VEGF_165a_ levels [64]. Thus, the blockade of SRPK1 could have therapeutic benefits in cancer treatment as it lowers a variant that promotes both sprouting and intussusceptive angiogenesis, namely, VEGF_165a_, without depleting VEGF_xxxb_. On the other hand, other studies have focused on VEGF_xxxb_, an isoform that halts tumor growth in several pre-clinical models. Rennel et al. [92] investigated the ability of VEGF_165b_ transfected and non-transfected cells to induce Ewing sarcoma and renal cell carcinoma. Interestingly, tumorigenesis was suppressed in mice injected with cells overexpressing VEGF_165b_. The isoform also reduced the ability of VEGF_165a_ to induce angiogenesis by blocking its binding to VEGFR-2 and thus inhibiting the phosphorylation of the receptor [60]. The administration of VEGF_165b_ in a mouse xenograft of human breast cancer effectively reduced angiogenesis and tumor growth [81]. Varey et al. showed further that colon cancer cells overexpressing VEGF_165b_ limited the tumor growth in mouse xenografts [93]. Similarly, Rennel and colleagues observed reduced growth of prostate cancer due to the presence of VEGF_165b_ overexpressing cells [92]. Moreover, metastatic colorectal cancer patients with a low VEGF_165b_: total VEGF ratio were found to respond better to bevacizumab than those with a high ratio [93]. These studies signify a possible therapeutic role for VEGF_165b_ in antiangiogenic approaches. In another study involving breast cancer patients, it was observed that following adjuvant therapy there was prolonged disease-free survival (DFS) and the VEGF_165b_ levels remained elevated even after 2 years [94]. The observations from the various studies have shed light on the effects of interventions employing VEGF_xxxb_, nonetheless, additional investigations using larger sample sizes are required. Other regulators of VEGF splicing, pH, and hypoxia can also be modulated to inhibit the formation of certain isoforms. For example, the stabilization of VEGF mRNA was achieved following the use of anisomycin [47]. This approach can be employed to counter VEGF splicing in response to changes in the milieu of tumor cells, such as decreasing pH or reducing the development of hypoxia. Furthermore, the drug abexinostat which targets molecules that promote HIF expression could be useful in suppressing hypoxia. Recently, the drug showed promising results in relapsed lymphoma [95]. Panobinostat, which also suppresses hypoxia, may also have potential application as part of a combination strategy with treatments that splice variants or their receptors. The drug was evaluated in Phase II clinical trials for the treatment of B-cell lymphoma and showed promising results [96]. It is also plausible that abexinostat and panobinostat could alleviate the development of resistance to therapies targeting VEGF splicing or its splice products.

## 5. Conclusions

To date, several anti-angiogenics have been designed to target VEGF and its receptors for cancer treatment. The prevailing theory was that such drugs would not lead to the development of resistance since the target, namely the endothelial cell, was genetically stable. However, the therapeutic benefits of these drugs have been modest and transient, and toxicity and refractory disease remain significant drawbacks. Historically, the stimulation of alternate VEGF-independent proangiogenic pathways was thought to promote the resumption of angiogenesis. In recent years, the existence of VEGF isoforms which are expressed differentially in various tissues and cancers has been reported. Furthermore, some of the variants trigger intussusceptive angiogenesis, a form of vascularization used by tumors to escape anti-angiogenic therapy. Given the distinct roles of VEGF variants in both angiogenesis modulation and neoplastic progression, it is plausible that they may act as contributors to the poor clinical outcome observed with current VEGF-targeting drugs. Moreover, these drugs target the variants indiscriminately, regardless of whether they contribute to angiogenesis or diminish the angiogenic response. It is evident from various studies that the balance between VEGF_xxxa_ and VEGF_xxxb_ is central in angiogenesis, and any disturbance of this balance alters the physiology of VEGF/VEGFR signaling. It is thus important to determine the profile of the VEGF transcripts in various cancers. Additionally, the receptors through which these ligands transduce signals also undergo splicing. While data are still sparse on the clinical impact of splice products of canonical VEGF receptors in cancer, their clinical significance mandates further exploration to improve treatment approaches. Concerning VEGF isoforms, it has been shown unequivocally that VEGF_165a_ promotes both sprouting and intussusceptive angiogenesis. Given the importance of SRPK in the formation of the VEGF_165a_ variant, the compound TG0003 which inhibits SRPK could be employed in order to lower VEGF_xxxa_ and restore the VEGF_165a_/VEGF_165b_ levels. On the other hand, VEGF_165b_ appears to have a dual effect, namely, the stimulation of angiogenesis, albeit weakly, and the competitive inhibition of VEGF_165a_ binding to VEGFR-2. Splice modifiers that can alter the outcome of pre-mRNA splicing may prove useful in restoring a disturbance in the VEGF_xxxa_/_xxxb_ ratio. Furthermore, drug carriers and antibodies could improve precision targeting and thus alleviate undesirable off-target effects observed with VEGFR inhibitors. Moreover, metallic nanoparticles, including gold nanoparticles, have been shown to be effective carriers in targeting tumor vessels in preclinical models. Combinations of carriers with inhibitors that target the overexpressed isoform or exogenous supplementation of the under-expressed VEGF_xxxb_ isoform could establish a healthy balance and thus benefit strategies that are geared towards anti-angiogenesis. Future studies should establish whether the restoration of the VEGF_xxxa/xxxb_ balance leads to vascular normalization, which is a desirable effect to enhance drug extravasation into the tumor and thus improve the effectiveness of chemotherapy and immune-modulating drugs.

## Figures and Tables

**Table 1:** VEGF variants that are commonly expressed in human cancers.

Variant	Receptor	Mechanism	Effect	Expression in Cancer	Reference
111a	VEGFR-2 VEGFR-1	VEGFR-2 phosphorylation; ERK 1/2 activation	Neovessel formation	↑NSCLC ↑Breast cancer ↑Ovarian cancer	[40,42]
121a	VEGFR-2 VEGFR-1	VEGFR-2 phosphorylation at Y1175; P13k/p38, MEK1-ERK1/2 activation	EC proliferation, tube formation in matrigel plug; regulates vessel diameter; vessel maturation	↑Prostate cancer ↑Colorectal cancer ↑Breast cancer	[43,64,65,66,67]
145a	VEGFR-2		EC mitogen; induces angiogenesis		[68]
165a	VEGFR-2 VEGFR-1	VEGFR-2 phosphorylation at Y1175 PIP2 hydrolysis and formation of IP3, activation of PKC	EC proliferation, migration, sprout formation; regulates vessel diameter; vessel maturation	↑Colorectal cancer ↑Cervical cancer ↑Esophageal cancer	[40,41,42,43,49,67]
165b	VEGFR-2 VEGFR-1	Incomplete phosphorylation at Y1175; No PKC activation/ PIP2 hydrolysis	Weakly angiogenic; competitively inhibits VEGF- VEGFR-2 binding	↓Breast cancer ↓RCC ↑Melanoma	[51,59,60]
189a	VEGFR-2 (weak)	Binds NRP-1; upregulates Flk-1	Cancer cell proliferation, EC proliferation, chemotaxis, tube formation		[69,70,71]
